# Development and Validation of Accelerometer-Based Machine Learning Models for Classifying Walking, Running, and Jumping Activities

**DOI:** 10.3390/s26092810

**Published:** 2026-04-30

**Authors:** Lucas Veras, Florêncio Diniz-Sousa, Giorjines Boppre, Ana Resende-Coelho, José Oliveira, Hélder Fonseca

**Affiliations:** 1 Research Center in Physical Activity, Health and Leisure (CIAFEL), Faculty of Sport, University of Porto, 4200-450 Porto, Portugalhfonseca@fade.up.pt (H.F.); 2 Laboratory for Integrative and Translational Research in Population Health (ITR), University of Porto, 4200-450 Porto, Portugal; 3 Institute of Molecular Sports and Rehabilitation Medicine, Paracelsus Medical Private University, 5020 Salzburg, Austria; 4 Centro Regional de Estudios Avanzados en Estilos de Vida Activos y Saludables (CREA-EVAS), Universidad Adventista de Chile, Chillán 3780000, Chile

**Keywords:** accelerometer, activity recognition, mechanical loading, validation, machine learning

## Abstract

Quantifying mechanical loading during daily physical activities is essential for designing and evaluating bone health interventions. Accelerometers are a promising tool for estimating these loads under free-living conditions, yet existing prediction models depend on prior knowledge of the activity being performed. This study developed and validated machine learning models to automatically distinguish between walking, running, and jumping using accelerometer data. Forty-eight healthy adults completed a protocol of walking, running, and jumping tasks while wearing ActiGraph GT9X Link accelerometers at the ankle, lower back, and hip. Three algorithms (Random Forest, Support Vector Machine, and K-Nearest Neighbors) were trained and evaluated through multiple performance metrics. All models achieved excellent classification accuracy across sensor placements, with percent agreement between 93.8% and 97.7%, receiver operating characteristic area under the curve values consistently above 0.97, and Kappa coefficients exceeding 0.89. These results demonstrate that accelerometer-based activity classification can reliably differentiate walking, running, and jumping, establishing a practical framework for applying activity-specific mechanical loading prediction equations under free-living conditions.

## 1. Introduction

Bone tissue adapts to mechanical loading by modifying its mass, geometry, and microarchitecture to optimize its strength and resistance to fracture [1,2,3]. Weight-bearing physical activities that generate ground reaction forces through impact with the ground, particularly walking, running, and jumping, have been consistently shown to promote positive skeletal adaptations and are widely prescribed to enhance bone health [4,5]. The osteogenic effects of these impact-generating activities are primarily mediated by specific mechanical loading characteristics, especially the magnitude and rate of the applied forces, which trigger bone’s adaptive response [3,6,7].

Accurately measuring these forces during daily activities and exercise is crucial for developing and monitoring effective bone health interventions [8]. While force plates remain the gold standard for measuring ground reaction forces [9], accelerometers have emerged as a promising alternative for measuring mechanical loading in free-living conditions [10,11,12,13]. However, current accelerometer-based prediction models typically require prior identification of the type of activity being performed, as different movement patterns produce distinct force profiles that necessitate specific prediction models [11,13,14,15,16]. This activity-dependent approach limits the practical application of these models in real-world settings, where individuals perform various activities throughout the day without systematic classification.

A potential solution to overcome this limitation is the use of models to automatically identify and classify activities from accelerometer data [17,18]. While various activity recognition approaches have been developed and validated in the literature, they typically focus on distinguishing between broad categories of movement (e.g., sitting, standing, walking) or common activities of daily living (e.g., household chores, stair climbing) [19,20]. Although some of these studies include walking and running among their classified activities [17,19,21], they were not specifically designed to differentiate between the types of movements most relevant for mechanical loading and bone adaptation [4,5]. Moreover, none of these approaches incorporate jumping activities, which are among the most osteogenic forms of exercise, and their broader classification scope may compromise the accuracy needed for activity-specific mechanical loading prediction. Such a targeted classification model would enable the appropriate application of activity-specific prediction equations, thereby improving the accuracy of mechanical loading assessment in free-living conditions. Therefore, this study aimed to develop and validate classification models capable of accurately differentiating between walking, running, and jumping activities using only accelerometer data, to enable the appropriate application of activity-specific mechanical loading prediction equations.

## 2. Materials and Methods

### 2.1. Participants

A total of 48 participants (26 males, 22 females) were recruited via convenience sampling for this study. Sample characteristics were ($\bar{X}$ ± SD): age (29.0 ± 8.3 years), height (169.3 ± 8.7 cm), body mass (69.7 ± 11.6 kg), and body mass index (24.2 ± 3.0 kg·m^−2^). Study recruitment was conducted through email distribution within the University of Porto’s academic network, detailing study aims, experimental procedures, and participation requirements. Physical measurements were obtained using standardized equipment: height was measured with a Seca stadiometer (model 213, Hamburg, Germany), while body mass was determined using a Seca digital weighing scale (model 899, Hamburg, Germany). To be eligible for participation, individuals needed to be between 18 and 65 years old and physically capable of completing the experimental protocol, which encompassed walking, running, and jumping tasks. Individuals were excluded if they reported any musculoskeletal disorders, cardiovascular conditions, or other health issues that could compromise their ability to safely perform the required activities. Prior to participation, all volunteers received detailed information about the study procedures and provided their written informed consent. The research protocol received approval from the local Ethics Committee (reference: CEFADE 15-21).

### 2.2. Protocol

The experimental procedures were performed at LABIOMEP-UP (Porto Biomechanics Laboratory). Participants were equipped with three ActiGraph GT9X Link accelerometers (ActiGraph, Pensacola, FL, USA), configured at 100 Hz sampling frequency with a ±16 g range (where 1 *g* equals 9.807 m·s^−2^). Each of the devices was placed on a different body position: one on the anterior axillary line at iliac crest height (right hip), another at the midpoint between the posterior superior iliac spines (lower back), and a third just above the lateral malleolus (right ankle). The selection of waist-level positions (hip and lower back) was based on their proximity to the body’s center of mass, enabling comprehensive movement assessment. To ensure proper device fixation, the waist-mounted accelerometers were attached using clips on an elastic belt, while the ankle unit was secured using a combination of adhesive tape and an elastic strap. Research staff personally supervised and verified the correct positioning and orientation of all devices on each participant.

The GT9X Link devices contain two triaxial accelerometers: a primary sensor with ±8 g range and proprietary filtering, and a secondary sensor with ±16 g range providing unfiltered data. For this study, we exclusively utilized data from the secondary accelerometer to maximize measurement precision and study reproducibility by avoiding potential filtering effects. Raw acceleration data along the *x*, *y*, and *z* axes were processed and extracted using ActiLife software (version 6.13.3; ActiGraph, Pensacola, FL, USA). All acceleration measurements were expressed in gravitational units.

The experimental protocol consisted of three main activities: walking (at normal, slow, and fast self-selected speeds), running (at slow and fast self-selected speeds), and various jumping tasks (including drop jumps, box jumps, countermovement jumps, horizontal jumps, side hops, and continuous jumps). Walking and running activities were performed on a 10 m path marked on the laboratory floor. Each participant performed multiple trials of each activity to ensure robust data collection, completing 9 trials for walking, 6 for running and 22 for jumping. Participants were instructed to perform all movements naturally, and adequate rest periods were provided between trials. Detailed description of the complete experimental protocol, including specific rest intervals, number of repetitions, and execution procedures can be found elsewhere [22].

To facilitate activity labeling during data processing, a video camera was positioned perpendicular to the movement path, at the midpoint of the laboratory’s main corridor at about 100 cm from the floor. This fixed camera position provided a sagittal plane view of the main laboratory path, allowing clear identification and verification of activity types and transitions during the data processing. Views from this camera can be seen in the Video S1.

### 2.3. Data Processing

The activity data labeling process was performed using custom MATLAB scripts (version 2024b; MathWorks, Natick, MA, USA). This procedure involved visual inspection of the acceleration signals alongside video recordings. The video footage, captured from the fixed camera position in the laboratory’s main corridor, served as a reference to accurately identify and verify each activity bout, as mentioned previously. Researchers manually identified the beginning and end of each activity segment using an interactive graphical interface that displayed the resultant acceleration signal, with the video timestamps helping to ensure precise temporal segmentation of the activities. For the walking and running activities, only data from the middle 4 m of the path were used to exclude the acceleration and deceleration periods. The segmented data were then exported as separate files containing the raw triaxial acceleration values (*x*, *y*, and *z* axes) for each activity trial.

Feature extraction from the accelerometer signals was performed using the R software (version 4.3.2; R Foundation for Statistical Computing, Vienna, Austria). For each activity trial, both time- and frequency-domain features were computed using a 1 s sliding window of the triaxial acceleration data. For each acceleration axis (*x*, *y*, z), time-domain features included basic statistical measures (mean, standard deviation, coefficient of variation), distribution characteristics (skewness, kurtosis), percentile values (minimum, 25th percentile, median, 75th percentile, maximum), and amplitude-related metrics (mean and standard deviation of the signal magnitude). Similarly, frequency-domain features were calculated independently for each axis using Fast Fourier Transform (FFT), comprising dominant frequency, dominant magnitude, total power, and median frequency. Additionally, cross-axis correlations were computed between all pairs of axes (*xy*, *xz*, *yz*). Lastly, for the orientation features (roll, pitch, yaw), the acceleration signals were first preprocessed using a 2nd-order Butterworth low-pass filter with a 1 Hz cutoff frequency, implemented using zero-phase digital filtering to prevent phase distortion. This process resulted in a comprehensive set of 54 features per activity segment that captured both the temporal and spectral characteristics of the movements.

### 2.4. Statistical Analyses

Prior to implementing the machine learning algorithms, we conducted exploratory data analysis using t-Distributed Stochastic Neighbor Embedding (t-SNE) to examine the structure of our high-dimensional dataset. This analysis was specifically performed to investigate whether the accelerometer data naturally clustered by activity type or if there was substantial participant-dependent variability that might influence the classification results.

After that, three supervised machine learning algorithms were implemented to classify the activities: Random Forest (RF), Support Vector Machine (SVM) with radial basis function kernel, and K-Nearest Neighbors (KNN). The RF algorithm is an ensemble learning method that combines multiple decision trees to handle complex movement patterns [23]. SVM with radial basis function kernel creates optimal boundaries to separate different activity classes [24]. KNN classifies activities based on their similarity to known samples [25]. These algorithms were selected for their strengths in handling non-linear relationships, creating clear activity distinctions, and recognizing movement patterns in accelerometer data [26].

The dataset was split into training (80%) and testing (20%) sets at the participant level, using stratified sampling to maintain class proportions, and ensuring that no participant’s data appeared in both sets. Prior to model training, features were preprocessed through standardization (centering and scaling of all predictors) and removal of predictors with near-zero variance (variables that showed minimal variation across observations and thus provided little discriminative value). Model hyperparameters were optimized using a grid search approach with 5-fold cross-validation on the training set. For the RF model, we tuned the number of randomly selected predictors (mtry), and the minimum number of data points required for a node split (min_n), while fixing the number of trees at 500. The SVM model’s cost parameter and kernel bandwidth (rbf_sigma) were tuned, while for the KNN model, the number of neighbors was optimized. For each model, 10 different hyperparameter combinations were evaluated. The hyperparameters used in the final models can be seen at the Supplementary Table S1.

Model performance was assessed using multiple metrics: percent agreement, area under the receiver operating characteristic curve (ROC-AUC), and Cohen’s Kappa coefficient. These metrics were calculated both for overall classification performance and for each activity individually using a one-versus-all approach. To identify which accelerometer-derived features were most influential in distinguishing between activities, we conducted a feature importance analysis using the RF algorithm’s permutation importance method. This approach measures each feature’s contribution to the classification task by quantifying the decrease in model performance when each feature is randomly shuffled, providing insights into which movement characteristics were most crucial for activity recognition. All analyses were performed separately for each accelerometer placement (ankle, lower back and hip) using R (version 4.5.1) with the tidymodels framework for machine learning (version 1.3.0).

## 3. Results

**Figure 1:**
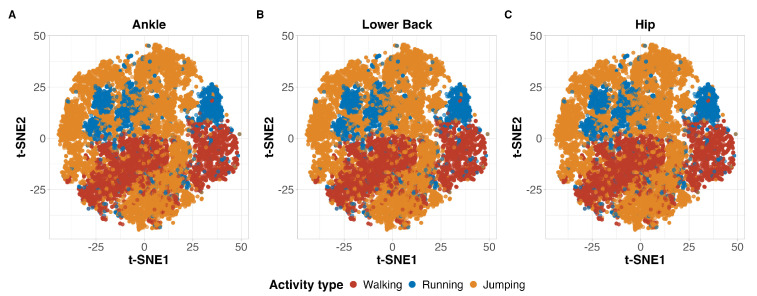
t-Distributed Stochastic Neighbor Embedding (t-SNE) visualization of accelerometer data showing consistent clustering of walking, running, and jumping activities across ankle (panel (**A**)), lower back (panel (**B**)), and hip (panel (**C**)) accelerometer placements.

The t-SNE visualization shown in Figure 1 revealed consistent clustering patterns across all three accelerometer placements, with walking, running, and jumping activities forming distinct clusters regardless of sensor location. All placements showed clear separation between activities, particularly for walking, with some expected overlap between running and jumping clusters, demonstrating that the inherent structure of these movement patterns was captured similarly whether the accelerometer was placed on the ankle, lower back, or hip.

All three machine learning algorithms demonstrated excellent classification performance across all accelerometer placements (ankle, lower back, and hip) and activities (walking, running, and jumping). The classification accuracy metrics with estimates of uncertainty, calculated using the 5-fold cross-validation in the training dataset, can be seen in Table 1. This analysis revealed high accuracy across all models and accelerometer placements, with percent agreement ranging from 91.6% to 95.6%, ROC-AUC from 0.980 to 0.992, and Kappa from 0.853 to 0.925, all with notably small uncertainty measures, demonstrating the robust and consistent performance of the classification approach.

Classification accuracy metrics in the testing dataset for each model across all accelerometer placements can be found on Table 2. The overall percent agreement ranged from 93.8% to 97.7%, with ROC-AUC values consistently above 0.97 and Kappa coefficients exceeding 0.89, indicating robust classification capability regardless of the specific algorithm or sensor placement chosen.

The ankle-mounted accelerometer demonstrated marginally superior overall performance, with KNN achieving the highest accuracy (97.7% agreement, ROC-AUC = 0.991, κ = 0.961), followed by RF (96.8% agreement, ROC-AUC = 0.996, κ = 0.945), and SVM (95.9% agreement, ROC-AUC = 0.996, κ = 0.930). For the lower back placement, RF showed the best performance (95.9% agreement, ROC-AUC = 0.994, κ = 0.930), followed closely by SVM (95.7% agreement, ROC-AUC = 0.993, κ = 0.926), and KNN (93.8% agreement, ROC-AUC = 0.971, κ = 0.895). Hip-mounted accelerometers maintained similar high-performance levels, with SVM achieving the highest accuracy (95.3% agreement, ROC-AUC = 0.993, κ = 0.920), followed by RF (94.7% agreement, ROC-AUC = 0.989, κ = 0.909), and KNN (94.0% agreement, ROC-AUC = 0.981, κ = 0.899).

Table 3 contains the same classification performance metrics, calculated in the testing dataset, for each model across all accelerometer placements, but specifically for each activity. When examining specific activities, all three algorithms demonstrated great performance in classifying walking activities. Very high and similar accuracy was achieved by KNN with ankle placement (98.2% agreement, ROC AUC = 0.995, κ = 0.960) and RF with lower back placement (98.4% agreement, ROC AUC = 0.999, κ = 0.965). SVM showed consistent performance across all placements, with agreement rates around 97%.

Running activities were also classified with high accuracy across all algorithms and placements with KNN with ankle placement showing the best performance (98.7% agreement, ROC AUC = 0.985, κ = 0.943). Random Forest and SVM demonstrated similar performance levels, with agreement rates ranging from 96.0% to 97.7%. The hip placement showed slightly lower but still acceptable performance compared to ankle and lower back placements.

Jumping activities were also classified with high accuracy, particularly by the ankle-mounted accelerometer. KNN achieved the highest accuracy with ankle placement (98.6% agreement, ROC AUC = 0.996, κ = 0.972). Random Forest and SVM showed strong performance as well, with agreement rates consistently above 95%. The hip placement demonstrated slightly lower accuracy compared to ankle and lower back placements, but still maintained agreement rates above 94%. These consistently high accuracy levels across all activities and placements suggest that these models could be reliably implemented in real-world applications for activity recognition and monitoring.

Analysis of the confusion matrices revealed that each algorithm demonstrated highly consistent classification patterns across all three accelerometer placements, as can be seen in Table 4. The RF models showed very similar performance for all placements, correctly classifying 480 walking instances (97.8% of the cases), 163 (78.4%) running instances, and 799 (97.1%) jumping instances, with comparable misclassification patterns (39 running instances misclassified as jumping, 14 jumping instances as running). Similarly, SVM maintained consistent performance across all placements, with 480 (97.8%) correct walking classifications, 185 (88.9%) correct running classifications, and 786 (95.5%) correct jumping classifications in each case. The KNN algorithm, which also showed very similar results regardless of placement, correctly identified 482 (98.2%) walking instances, 185 (88.9%) running instances, and 764 (92.8%) jumping instances at all locations. Across all algorithms and placements, walking activities showed the highest classification stability with minimal misclassifications, while most classification errors occurred in distinguishing between running and jumping activities. This highly consistent performance across placements suggests that the classification challenges were inherent to the movement patterns rather than sensor location-dependent.

**Figure 2:**
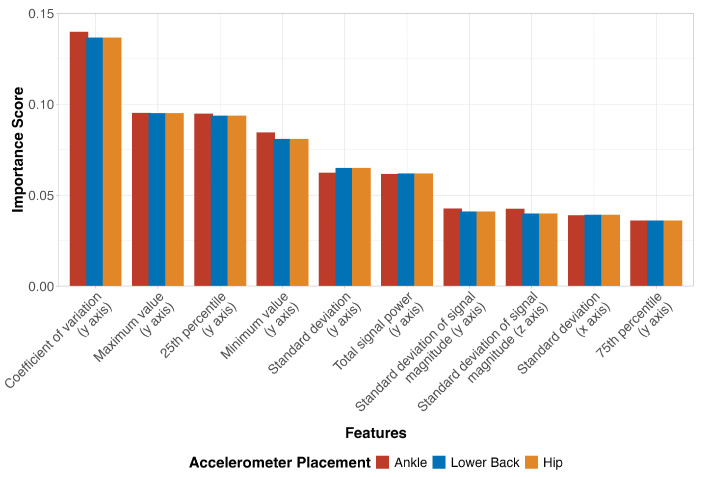
Feature importance scores derived from random forest models for ankle, lower back, and hip accelerometer placements. The importance scores indicate the relative contribution of each feature to the classification of walking, running, and jumping activities, with higher scores representing greater importance for activity discrimination.

Figure 2 shows the feature importance analysis using the RF algorithm. This analysis revealed a consistent pattern of influential features across all accelerometer placements. Vertical (*y*) axis characteristics dominated the top-ranked features, with coefficient of variation showing the highest importance score across all placements, followed by maximum value and 25th percentile measures. Signal magnitude characteristics and standard deviation measures from multiple axes also emerged as important discriminative features, though with lower importance scores. This hierarchy of feature importance was remarkably similar across ankle, lower back, and hip placements, indicating that vertical movement patterns, particularly their variability and peak characteristics, provide the strongest discriminative information for classifying walking, running, and jumping activities, regardless of sensor location.

To ensure reproducibility and facilitate implementation of our findings, both the feature extraction code and the trained classification models have been made publicly available at https://github.com/verasls/activity_recognition (accessed on 25 April 2026). The repository includes detailed documentation for implementing the processing pipeline and using the pre-trained models for activity classification.

## 4. Discussion

This study aimed to develop and validate classification models capable of accurately differentiating between walking, running, and jumping activities using accelerometer data, to enable the appropriate application of activity-specific mechanical loading prediction equations. Three supervised machine learning algorithms (RF, SVM, and KNN) were implemented and validated using multiple performance metrics. Our results demonstrated that all three algorithms achieved excellent classification accuracy across all accelerometer placements and activities, with percent agreement ranging from 93.8% to 97.7%, ROC-AUC values consistently above 0.97, and Kappa coefficients exceeding 0.89. This robust classification performance was supported by t-SNE visualization, which revealed consistent clustering patterns of the three activities across all accelerometer placements. The ankle-mounted accelerometer showed marginally superior overall performance, with KNN achieving the highest accuracy, though all placements and algorithms demonstrated robust classification capability. These findings indicate that accelerometer-based activity classification is a reliable approach for distinguishing between walking, running, and jumping activities, providing a foundation for the accurate application of activity-specific mechanical loading prediction equations. Our classification models demonstrated remarkably high accuracy across different accelerometer placements and activities, with only marginal differences between algorithms. The RF, SVM, and KNN algorithms all achieved overall agreement rates above 93%, with minimal variations in performance that are not practically meaningful. All machine learning models achieved slightly superior results using ankle-worn accelerometers, with a 97.7% percent agreement for KNN, 96.8% for RF and 95.9% for SVM. These minimal differences suggest that any combination of algorithm and placement could be effectively implemented.

When analyzing specific activities, walking classifications showed the highest stability across all algorithms and placements, with agreement rates consistently above 95%. Running activities were classified with similarly high accuracy, particularly by KNN with ankle placement (98.7% agreement), though the hip placement showed marginally lower performance compared to ankle and lower back placements. For jumping activities, while maintaining high accuracy levels above 94% across all conditions, the ankle-mounted accelerometer demonstrated particularly strong performance, with KNN achieving 98.6% agreement. The consistency in performance across different placements suggests that the classification challenges were inherent to the movement patterns rather than being sensor location-dependent [20,27]. This was expected, as different movement patterns produce distinct force and acceleration profiles, which is why mechanical loading prediction models are typically activity-specific [14,28]. While the ankle placement showed a slight advantage in overall accuracy, these differences were not substantial enough to preclude the use of any placement location.

Examining the performance by activity type, our models showed consistently high accuracy across all activities, with only minor variations between them. Walking was very accurately classified across all models and placements, with percent agreement ranging from 95.7% to 98.4% and ROC-AUC values above 0.992. This high accuracy for walking can be attributed to its distinctive rhythmic pattern and acceleration magnitude that clearly differentiates it from both static activities and higher-intensity movements [29]. Running classification showed similarly high accuracy, with percent agreement ranging from 96.0% to 98.7% across placements and models. The main challenge in running classification appeared in its occasional misclassification as jumping, particularly when using the hip placement (14 to 24 misclassified instances). This confusion likely occurs due to the similar impact characteristics of these activities, such as their high magnitude and frequency [15,16,20]. Jumping activities also showed high classification accuracy (94.6% to 98.6% agreement). The confusion matrices reveal that most misclassifications occurred between running and jumping activities, with the number of misclassified instances between these two activities ranging from 14 to 39 depending on the algorithm. This corroborates what was observed in the t-SNE visualization, which showed some degree of overlap between the running and jumping clusters. The SVM algorithm showed the fewest total misclassifications between these two activities (32 instances combined), compared to KNN (38 instances) and RF (53). This misclassification pattern was consistent across all accelerometer placements, suggesting that the challenge lies in the inherent similarity of the movement patterns rather than sensor location [30]. The ankle placement showed marginally better discrimination between jumping and running activities, likely due to an ability to better capture the distinct landing mechanics of jumps versus running strides [31]. The feature importance analysis supported the consistently high accuracy across activities found in this study. It revealed that vertical acceleration characteristics, particularly coefficient of variation, maximum values, and percentile measures, were the most influential features regardless of the accelerometer placement. It also may explain why activities with similar vertical movement patterns, such as running and jumping, were occasionally misclassified.

Our results can be compared with several previous studies that used similar methodologies for activity classification. Bach et al. (2022) [19] used a dual accelerometer setup (lower back and thigh) with an extreme gradient boosting classifier to detect sitting, standing, lying, walking, running, and cycling during free-living conditions. Their dual-accelerometer setup achieved up to 96% accuracy, while their single thigh and back accelerometer setups achieved up to 93% and 84% accuracy, respectively. In comparison, our models achieved similar accuracies (93.8-97.7%) but with single accelerometer placements, suggesting that our approach might be more efficient as it eliminates the need for multiple sensors. Chong et al. (2021) [17] used a single hip-worn accelerometer to classify activities including lying, standing, walking, jogging, cycling, and various daily activities, testing artificial neural networks, SVM, and RF algorithms. For the hip placement, which allows direct comparison with our study, their models achieved accuracies between 86.9% and 88.0%, while our hip-placed models achieved 94.0-95.3% accuracy across all three algorithms, representing a consistent improvement. Nunavath et al. (2021) [21] employed deep learning approaches (deep feed-forward neural network and deep recurrent neural network) with both wrist-worn and hip-worn accelerometers to classify various activities including lying, sitting, standing, walking, running, and other daily activities. For the hip-worn setup classifying walking and running activities, they achieved accuracies between 84.89% and 89.31%, while our machine learning approaches achieved 94.0-95.3% accuracy for the same placement, suggesting that simpler algorithms might be more effective for this specific classification task.

The current study presents several key methodological strengths. First, while each model requires only a single accelerometer to function, we validated our approach across three different placements (ankle, lower back, and hip), achieving consistently high accuracy across all locations. This multi-placement validation is particularly valuable as it allows researchers to select the most appropriate placement for their specific needs [32], making our models compatible with existing datasets and studies that may have used different sensor locations. Second, our focus on walking, running, and jumping activities was specifically designed to address the needs of bone health research, as these activities are consistently shown to promote positive skeletal adaptations and are widely prescribed in bone health interventions [4,33]. The high classification accuracy achieved for these particular activities (>94% agreement across all activities) enhances the models’ utility for monitoring and prescribing bone-targeted exercise interventions. Third, the robust performance across different machine learning algorithms (RF, SVM, and KNN) and accelerometer placements demonstrates the reliability and stability of our approach, with all combinations achieving above 93% agreement. Finally, to ensure practical implementation of our findings, we have made available both the feature extraction code and the trained classification models. This open availability of the complete analysis pipeline significantly facilitates the direct application of our methods in future research and clinical practice, addressing a common limitation in machine learning studies in which methods are often not readily implementable by other researchers.

The activity classification models developed in this study open important practical applications, particularly when integrated with previously developed mechanical loading prediction equations [10,11,13,15,16]. By accurately identifying specific activities, these models address a critical limitation in mechanical loading assessment, as applying equations without distinguishing between activity types can lead to significant prediction errors–for instance, using walking-based equations for running or jumping activities could substantially underestimate peak forces and loading rates due to the distinct biomechanical characteristics of each movement [34,35]. Our models enable the appropriate application of activity-specific equations to free-living data through automated activity classification, allowing accurate prediction of mechanical loading variables such as ground reaction forces and loading rates, which are key determinants of bone adaptation [36,37]. In practice, the proposed data analysis workflow involves applying the classification models developed in this study to automatically identify each data segment as walking, running, or jumping, and then applying the corresponding activity-specific mechanical loading prediction equations to each classified segment. Both the feature extraction code and trained classification models are publicly available at our repository to facilitate this implementation.

This integration provides a comprehensive method to monitor both the type and mechanical load characteristics of physical activities in free-living conditions. Such capability has significant implications for bone health monitoring, as it allows researchers and clinicians to precisely quantify the osteogenic potential of daily activities by combining information about movement patterns and their associated mechanical loads [38], for which there is currently no available tool. In exercise prescription and rehabilitation, this method could also help ensure that patients receive adequate bone-stimulating forces while avoiding excessive loads that might increase injury risk [39,40]. For instance, clinicians could monitor not only if a patient is performing the prescribed activities (e.g., walking versus running), but also track the cumulative mechanical loading throughout the day [41]. However, considerations for real-world implementation include the need for strategies for managing data collection and processing in large-scale studies, and the development of user-friendly interfaces for clinicians and researchers who may not have programming expertise.

Some limitations should also be acknowledged. First, while our sample size of 48 participants was adequate for model development and validation, it may not fully capture the variability in movement patterns across different populations. Future studies should validate these models in larger and more diverse cohorts, particularly including participants with greater variability in age, body mass, and pathologies, as these factors are known to influence model performance [42]. Second, although our protocol included various activities performed in a semi-controlled environment, it may not completely represent the complexity and variability of movements in free-living conditions. Despite instructing participants to perform activities naturally, the laboratory setting might have influenced their movement patterns. Additionally, free-living data present further challenges not addressed in the current study, including continuous unsegmented data streams, transitions between activities, and non-target movements (e.g., stair climbing, static postures, household tasks) that fall outside the current classification scheme. Therefore, future research should validate these models during extended periods of free-living activities and with the use of a more diverse set of activities to ensure their reliability in real-world conditions.

In conclusion, this study successfully developed and validated machine learning models capable of accurately classifying walking, running, and jumping activities using accelerometer data from a single sensor. The high classification accuracy achieved across different algorithms (RF, SVM, and KNN), accelerometer placements (ankle, lower back, and hip), and activities (walking, running and jumping) demonstrates the robustness and reliability of our approach. Overall, the choice of algorithm may depend on the intended application, as the KNN algorithm with ankle placement achieved the highest accuracy (97.7%), while the RF demonstrated the most consistent performance across all accelerometer placements. The consistent performance across different sensor locations provides researchers flexibility in choosing the most appropriate placement for their specific needs. When combined with previously developed mechanical loading prediction equations, these models enable the comprehensive assessment of both activity types and their associated mechanical loads during free-living conditions. This integration represents a significant advancement in the objective monitoring of bone-targeted exercise interventions, offering researchers and clinicians a practical tool for quantifying the osteogenic potential of daily activities.

## Figures and Tables

**Table 1:** Accuracy indices of machine learning algorithms for walking, running and jumping activities classification across different accelerometer placements. Metrics were calculated using the 5-fold cross-validation in the training dataset. Each metric is presented as mean ± standard error.

Machine Learning Algorithm	Accelerometer Placement	Percent Agreement	ROC-AUC	Kappa
Random Forest	Ankle	95.6 ± 0.1	0.992 ± 0.001	0.925 ± 0.001
	Lower Back	95.5 ± 0.3	0.992 ± 0.002	0.923 ± 0.005
	Hip	95.5 ± 0.3	0.992 ± 0.002	0.923 ± 0.005
Support Vector Machine	Ankle	91.6 ± 0.3	0.980 ± 0.001	0.853 ± 0.005
	Lower Back	91.7 ± 0.1	0.980 ± 0.003	0.854 ± 0.002
	Hip	91.7 ± 0.1	0.980 ± 0.003	0.854 ± 0.002
K-Nearest Neighbors	Ankle	93.9 ± 0.3	0.981 ± 0.003	0.897 ± 0.006
	Lower Back	93.8 ± 0.4	0.980 ± 0.002	0.895 ± 0.006
	Hip	93.8 ± 0.4	0.980 ± 0.002	0.895 ± 0.006

Abbreviation: ROC-AUC, receiver operating characteristic area under the curve.

**Table 2:** Accuracy indices of machine learning algorithms for walking, running and jumping activities classification across different accelerometer placements. Metrics were calculated using the testing dataset. Cell’s background is colored in grayscale, where darker cells represent higher accuracy values.

Machine Learning Algorithm	Accelerometer Placement	Percent Agreement	ROC-AUC	Kappa
Random Forest	Ankle	96.80%	0.996	0.945
	Lower Back	95.90%	0.994	0.93
	Hip	94.70%	0.989	0.909
Support Vector Machine	Ankle	95.90%	0.996	0.93
	Lower Back	95.70%	0.993	0.926
	Hip	95.30%	0.993	0.92
K-Nearest Neighbors	Ankle	97.70%	0.991	0.961
	Lower Back	93.80%	0.971	0.895
	Hip	94.00%	0.981	0.899

Abbreviation: ROC-AUC, receiver operating characteristic area under the curve.

**Table 3:** Accuracy indices of machine learning algorithms for walking, running and jumping activities classification across different accelerometer placements and activities. Metrics were calculated using the testing dataset. Cell’s background is colored in grayscale, where darker cells represent higher accuracy values.

Machine Learning Algorithm	Accelerometer Placement	Activity	Percent Agreement	ROC-AUC	Kappa
Random Forest	Ankle	Walking	98.00%	0.998	0.955
		Running	97.70%	0.995	0.902
		Jumping	97.80%	0.999	0.957
	Lower Back	Walking	98.40%	0.999	0.965
		Running	96.70%	0.989	0.855
		Jumping	96.70%	0.995	0.934
	Hip	Walking	98.20%	0.998	0.96
		Running	96.00%	0.983	0.82
		Jumping	95.30%	0.991	0.905
Support Vector Machine	Ankle	Walking	97.80%	0.998	0.95
		Running	97.30%	0.994	0.882
		Jumping	96.70%	0.998	0.934
	Lower Back	Walking	97.60%	0.996	0.946
		Running	97.20%	0.992	0.878
		Jumping	96.60%	0.993	0.931
	Hip	Walking	97.40%	0.997	0.942
		Running	97.30%	0.992	0.885
		Jumping	95.90%	0.992	0.918
K-Nearest Neighbors	Ankle	Walking	98.20%	0.995	0.96
		Running	98.70%	0.985	0.943
		Jumping	98.60%	0.996	0.972
	Lower Back	Walking	95.70%	0.978	0.904
		Running	97.20%	0.965	0.883
		Jumping	94.70%	0.969	0.894
	Hip	Walking	96.50%	0.992	0.922
		Running	96.90%	0.971	0.869
		Jumping	94.60%	0.982	0.892

Abbreviation: ROC-AUC, receiver operating characteristic area under the curve.

**Table 4:** Confusion matrix for activity classification for each machine learning algorithm, calculated using the testing dataset. Diagonals represent the correct classification and are highlighted in bold. All classification scores are highly consistent across all accelerometer placements within the same algorithm.

Machine Learning Algorithm	Predicted	Observed		
		Walking	Running	Jumping
Random Forest	Walking	**480 (97.8%)**	6 (2.9%)	10 (1.2%)
	Running	2 (0.4%)	**163 (78.4%)**	14 (1.7%)
	Jumping	9 (1.8%)	39 (18.8%)	**799 (97.1%)**
Support Vector Machine	Walking	**480 (97.8%)**	8 (3.8%)	20 (2.4%)
	Running	1 (0.2%)	**185 (88.9%)**	17 (2.1%)
	Jumping	10 (2.0%)	15 (7.2%)	**786 (95.5%)**
K-Nearest Neighbors	Walking	**482 (98.2%)**	9 (4.3%)	35 (4.3%)
	Running	0 (0.0%)	**185 (88.9%)**	24 (2.9%)
	Jumping	9 (1.8%)	14 (6.7%)	**764 (92.8%)**

Data shown as n (%). Percentage values were calculated per column.

## Data Availability

The datasets presented in this article are not readily available because the data are part of an ongoing study. Requests to access the datasets should be directed to Lucas Veras.

## Supplementary Materials

The following supporting information can be downloaded at: https://www.mdpi.com/article/10.3390/s26092810/s1. Video S1: View from the camera used in the protocol. Table S1: Model hyperparameters for each machine learning algorithm and accelerometer placement.

## Abbreviations

The following abbreviations are used in this manuscript:

KNN	K-nearest neighbors
RF	Random forest
ROC-AUC	Area under the receiver operating characteristic curve
SVM	Support vector machine
t-SNE	t-distributed stochastic neighbor embedding
