# Supplementary material for: Development and Validation of Accelerometer-Based Machine Learning Models for Classifying Walking, Running, and Jumping Activities
Source: Sensors (Basel). 2026 Apr 30;26(9):2810. doi: 10.3390/s26092810 (PMC13165600; doi:10.3390/s26092810)
Supplement: Supplementary file 1 [file sensors-26-02810-s001.zip › tableS1.pdf]

Table S1. Model hyperparameters for each machine learning algorithm and accelerometer placement.

Machine Learning Algorithm	Accelerometer Placement	Hyperparameters
Random Forest	Ankle	mtry = 18, min_n = 7
	Lower Back	mtry = 9, min_n = 9
	Hip	mtry = 37, min_n = 5
Support Vector Machine	Ankle	cost = 4.38, rbf_sigma = 0.108
	Lower Back	cost = 3.00, rbf_sigma = 0.027
	Hip	cost = 6.42, rbf_sigma = 0.010
K-Nearest Neighbors	Ankle	neighbors = 8
	Lower Back	neighbors = 2
	Hip	neighbors = 7
